# Surgical treatment indications and outcomes in patients with spinal metastases in the cervicothoracic junction (CTJ)

**DOI:** 10.1186/s13018-018-0732-2

**Published:** 2018-01-30

**Authors:** Zemin Li, Houqing Long, Rui Guo, Jinghui Xu, Xiaobo Wang, Xing Cheng, Yangliang Huang, Fobao Li

**Affiliations:** 1 Department of Spine Surgery, The First Affiliated Hospital of Sun Yat-sen University, Guangzhou, China; 2 Department of Radiation Oncology, Sun Yat-sen University Cancer Center, State Key Laboratory of Oncology in Southern China, Collaborative Innovation Center of Cancer Medicine, Guangzhou, China

**Keywords:** Spinal metastases, Cervicothoracic junction, Surgical treatment, Survival, Retrospective study

## Abstract

**Background:**

The cervicothoracic junction (CTJ) site accounts for approximately 10% of all spinal metastases. The complex anatomical and biomechanical features increase the difficulty in surgical treatment of the CTJ metastases. However, few studies in the literature on surgical treatment for spinal metastases are focusing on this special area. The aim of this study was to evaluate the surgical outcome of patients with CTJ metastases and analyze the prognostic factor for the postoperative survival.

**Methods:**

Total of 34 patients with CTJ metastases who underwent surgery in our department were retrospectively analyzed. We evaluated records for the details of medical history, treatment, surgery, radiographic imaging, and follow-up. Outcomes were assessed by overall survival as well as modified Tokuhashi score, SINS, Frankel grade, visual analog scale (VAS), and Karnofsky Performance Status (KPS).

**Results:**

The entire patients’ median survival time was 12.4 months (range, 3.5–36.2 months). Pain improved in 32 patients (94.12%), and the postoperative VAS scores were significantly improved compared with preoperative data. Majority of patients (71%) maintained or improved their Frankel scores 1 year after surgery. KPS scores improved in 13 patients (38%), remained stable in 19 (56%), and worsened in 2 (6%) postoperatively. Notably, patients with neurological deficit that did not improve after surgery had significantly worse median survival than those who had either no deficit or who improved after surgery. There were no instrumentation failures in this study.

**Conclusions:**

Surgical treatment is effective for patients of CTJ metastases, with a tolerable rate of complications. Remained or regained ambulatory status predicted overall survival. Thus, prompt and aggressive decompressive surgery is recommended for CTJ metastases patients with neurological impairment.

## Background

The spine is the most common site for bony metastases [1]. About 10–30% of the cancer patients are attained by spinal metastases [2, 3]. The incidence of spinal metastases continues to increase, likely a result of improvement in medical treatment and increasing survival times [2]. Among the adults, 60% of spinal metastases are either from breast, lung, or prostate cancer [4]. About one third of these spinal metastases become symptomatic, which is causing pain, neurological deficits, and biomechanical instability requiring surgical treatment [5]. The aim of surgery in such patients is to reduce neurological deficits and improve pain, thus improving the patient’s quality of life.

The cervicothoracic junction (CTJ) site accounts for approximately 10% of all spinal metastases [6]. The CTJ area has features in that the cervical lordosis merges into thoracic kyphosis in this region, and the lateral mass of C7 is transitional [7]. Furthermore, the complex biomechanics also increase the difficulty in surgical treatment of the CTJ metastases. However, few studies in the literature on surgical treatment for spinal metastases are focusing on this special area [7]. Therefore, the purpose of this study is to evaluate the outcomes of a cohort of patients undergoing surgery for the *cervicothoracic site* metastases.

## Methods

Approval of the institutional review board of our institution was obtained prior to the current study. Patients with spinal metastases or proposed surgery involving C7-T2 were included. There were 36 patients with CTJ metastases who underwent surgery in our department between May 2012 and December 2015. However, two patients lost their follow-up at 3 and 7 months after surgery respectively, and they were not included in this research. At last, a total of 34 patients were included and retrospectively analyzed in our study. The diagnosis of spinal metastasis was confirmed histologically, and diagnostic imaging including X-ray, computed tomography (CT), or magnetic resonance imaging (MRI), as well as PET-CT. The selection criteria for surgical intervention required the patients to have at least one of the following conditions: (1) Spine Instability Neoplastic Score (SINS) > 6, indicating spinal instability requiring surgical reconstruction; (2) significant or progressive neurological deficits/or no neurological recovery under 2-weeks’ non-surgical treatment; and (3) intractable pain under 2-weeks’ treatment of pain relief medication. Patients’ choice is also an important consideration, e.g., patients who rejected surgery were not included in our study. Patients excluded from surgery were those with an estimated survival less than 3 months or poor health situation to undergo surgery. All patients who underwent surgery have met the above indications.

The medical characteristics were retrospectively analyzed for demographic, clinical, radiographic, and histological data. The location of the spinal lesions was assessed using magnetic resonance (MR) imaging and plain X-rays. The modified Tokuhashi score and SINS were applied to evaluate the patients’ prognosis and spinal stability [8, 9]. The Frankel grading system and visual analog scale (VAS) were used to assess the neurological signs and the severity of pain [10, 11]. The patients’ life quality was assessed by Karnofsky Performance Status (KPS). The demographic and clinical characteristics of these patients are illustrated in Table 1. The operative data included surgical approach/procedure, blood loss, and perioperative complications. Blood loss included direct blood loss during surgery and blood loss until removing the tube.

**Table 1:** Demographics and clinical characteristics of study patients

Characteristics	*n* (%)
Age (years)	
Median	54
Range	34–72
Gender	
Male	17 (50)
Female	17 (50)
Primary tumors	
Lung	13 (38.2)
Breast	7 (21.9)
Prostate	6 (17.6)
Gastrointestinal	2 (5.9)
Other	6 (17.6)
Number of involved vertebrae	
1–2	26 (76.5)
> 2	8 (23.5)
Visceral metastases	
No	23 (67.6)
Yes	11 (32.4)
Frankel score at entry	
A, B, C	18 (52.9)
D, E	16 (47.1)
Modified Tokuhashi score	
0–8	22 (64.7)
9–12	12 (35.3)
SINS score	
7–12	23 (67.6)
13–18	11 (32.4)

### Surgical procedures

The aim of surgery was to provide immediate direct circumferential decompression of the spinal cord and reconstruction of the spinal stability. There were four surgeons involved in the surgery. Prof. HL performed all the cases as primary surgeon; Dr. ZL, YH, and JX were assistants during the surgeries. The surgical implants were chosen from different companies based on the operative strategies and surgical approaches (companies including Stryker Spine, MI, USA and DePuy Synthes Spine, MA, USA). The protocol did not specify operative techniques or fixation devices. The surgical strategy was determined for each patient depending on the involved level, tumor location, and the patient’s condition. In general, three approaches were applied in our study: (1) anterior approach: for anteriorly located tumors, e.g., tumors involving the vertebral body and/or encroached the spinal canal anteriorly, the approach was anterior; (2) posterior approach: mainly posteriorly located tumors, e.g., tumors mainly involving lamina, pedicle, spinous process, or other posterior elements, and/or encroached the spinal canal posteriorly or laterally, while the anterior structures were minor or not involved, a laminectomy and decompression were done and any other posterior elements involved were removed; and (3) combined posterior-anterior approach: tumors involving both the vertebral body and the lamina, or preexisting spinal deformity, a combined posterior-anterior approach was used. Fixation devices including screws, plate, titanium rods, mesh, or artificial vertebral body were used.

Intra-operative neuromonitoring (IOM) were applied during all the surgical procedures routinely using Xltek® Protektor32 IOM System (Natus Medical Incorporated, Ontario, Canada). Changes in transcranial motor-evoked potentials (tcMEPs) and somatosensory-evoked potentials (SSEPs) were monitored.

### Postoperative data

All patients received systemic tumor treatment after surgery. Patients were evaluated at the time of discharge and at postoperative time points of 1 month, 3 months, 6 months, and 1 year after surgery. Comparisons were made between preoperative and postoperative Frankel grade, VAS pain score, and KPS score. Postoperative spine MR images and X-rays were evaluated. Survival time after surgical treatment was also recorded.

### Statistical analysis

Postoperative survival as a function of time was expressed using Kaplan-Meier estimates with death as the failure event. Survival curves were compared using the Mantel-Cox test, and hazard ratios (HR) and 95% confidence intervals (CI) were computed. The Wilcoxon signed-rank test was used to compare non-parametric paired variables. Pearson correlation analysis was used to further evaluate the correlation between VAS and KPS scores. Statistical analyses were performed using GraphPad Prism 5.01 (GraphPad Software Inc., La Jolla, CA) and IBM SPSS 22.0 (SPSS Inc., Chicago, IL). For all analyses, probability values < 0.05 were considered statistically significant. Median values were reported with range.

## Results

### Surgical information of the patients

**Fig. 1:**
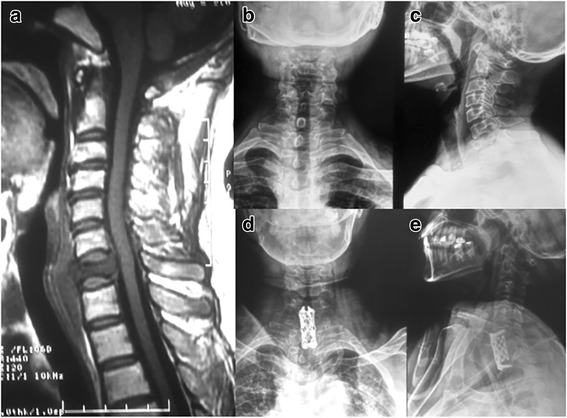
A 47-year-old female with lung cancer was admitted because of severe neck pain and upper extremity numbness. Mid-sagittal T1 MR image (**a**), anteroposterior (**b**), and lateral (**c**) radiographs showing C7 body pathological fracture and encroachment of spinal canal. Anteroposterior (**d**) and lateral (**e**) radiographs were obtained after C7 anterior cervical corpectomy and fusion (ACCF). The patient’s symptoms were significantly relieved

**Fig. 2:**
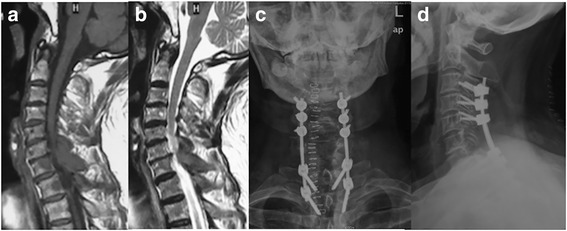
A 66-year-old male with prostate cancer was admitted because of severe neck pain and lower extremity weakness. Mid-sagittal T1 (**a**) and T2 (**b**) MR image showing multiple level lesions in the cervicothoracic junction area and posterior encroachment of spinal canal. Anteroposterior (**c**) and lateral (**d**) radiographs were obtained after posterior tumor resection, decompression, and fixation. The patient’s pain and neurological deficit were significantly improved

**Fig. 3:**
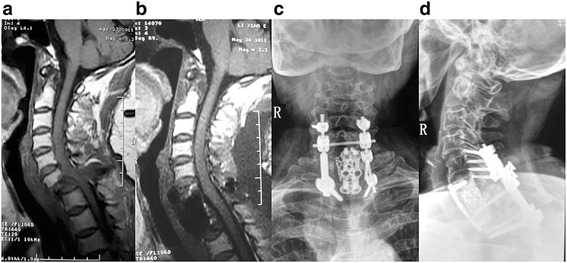
A 63-year-old female with breast cancer was admitted because of nonambulatory and severe pain. Mid-sagittal T1 MR image (**a**) showing C7 pathological fracture-dislocation and encroachment of spinal canal. Postoperative T1 MR image (**b**) obtained after combined posterior resection, anterior corpectomy, and reconstructive surgery, showing the spinal canal was significantly decompressed. Postoperative anteroposterior (**c**) and lateral (**d**) radiographs showing the good spinal alignment achieved after surgery. The patient regained ambulatory and self-care ability

The details of all surgical procedures are shown in Table 2. A total of 40 procedures were performed in 34 patients. Eight patients underwent anterior approach alone (Fig. 1); the median blood loss was 140 ml (range, 50–440 ml). Twenty-two patients underwent posterior approach alone (Fig. 2); the median blood loss was 320 ml (range, 100–970 ml). Four patients underwent combined posterior-anterior approach (Fig. 3); the median blood loss was 580 ml (range, 530–1100 ml). Blood loss was significantly higher in the combined approach compared with either the anterior or posterior approaches alone (*p* = 0.004 and 0.005, respectively).

**Table 2:** Surgery and perioperative procedures of study patients

Related procedures	*n* (%)
Anterior approach	8 (23.5)
Posterior approach	22 (64.7)
Combined posterior-anterior approach	4 (11.8)
Median blood loss (ml), (range)	315 (50–1100)
Instrumented spinal levels	
≤ 4	9 (26.5)
> 4	25 (73.5)
Complications	4 (11.8)
Deep wound infection	1 (2.9)
Acute epidural hematoma	1 (2.9)
Cardio-respiratory worsening	1 (2.9)
Cerebrospinal fluid leakage/effusion	1 (2.9)

### Pain status

**Fig. 4:**
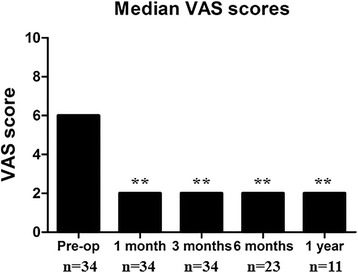
Pre- and postoperative median VAS scores during 1 year of follow-up, statistically significant at all time points. (** *P* < 0.001)

Overall, pain improved in 32 patients (94.12%), remained unchanged in 2 (5.88%), and worsened in 0 (0%) patients. A comparison of preoperative and postoperative median VAS scores is illustrated in Fig. 4. The preoperative median VAS was six, whereas the postoperative median VAS was two. This was significantly lower than preoperative pain scores at all time points (*p* < 0.001).

### Neurological status

The Frankel scores improved in 15 patients (44%), remained stable in 19 (56%), and deteriorated in 0 (0%). Eighteen patients (53%) were ambulatory at presentation (Frankel D-E) and 22 (65%) remained stable or improved. Of the 16 patients (47%) who were nonambulatory (Frankel A-C) preoperatively, 6 of them regained ambulatory. Postoperative Frankel scores of patients during follow-up is shown in Table 3. Overall, the majority of patients (71%) maintained or improved their Frankel scores immediately and up to 1 year after surgery.

**Table 3:** Frankel score over time

		Time from surgery				
		Pre-op *n* (%)	1 month *n* (%)	3 months *n* (%)	6 months *n* (%)	1 year *n* (%)
Frankel score	E	4 (12)	11 (32)	11 (32)	7 (29)	4 (33)
	D	12 (35)	11 (32)	13 (38)	13 (54)	7 (58)
	C	14 (41)	10 (29)	9 (26)	4 (17)	1 (8)
	B	3 (9)	2 (6)	1 (3)	0 (0)	0 (0)
	A	1 (3)	0 (0)	0 (0)	0 (0)	0 (0)
Total		34 (100)	34 (100)	34 (100)	24 (100)	12 (100)

### Functional status

With regard to functional outcomes, 17 patients presented with a KPS ≥ 70 (50%), and 17 patients demonstrated a preoperative KPS < 70 (50%). Overall, KPS scores improved in 13 patients (38%), remained stable in 19 (56%), and worsened in 2 (6%) postoperatively.

### Changes in IOM

Most of the patients (25/34) did not show any change in tcMEPs during surgery. One patient with a pre-op Frankel E showed a lasting amplitude drop of more than 50% from baseline in tcMEPs (both lower extremities) when underwent decompression procedure. After a careful check of the anatomy and instrumentation, we decided to continue the surgery cautiously. The patient showed a deterioration in neurological function (Frankel D) of both lower extremities immediately after surgery; however, he recovered (Frankel E) at the first day after surgery. Of the eight patients who showed improvement in voltage and/or amplitude of tcMEPs, seven patients (87.5%) improved their Frankel grade and six patients (75%) improved their KPS score. As regard to SSEPs, 21 patients did not show any change, while three patients showed SSEP deterioration (more than 50% drop in amplitude) and then subsequently recovered during the surgical procedure. Of the ten patients who showed improvement in SSEPs, eight patients (80%) improved their Frankel grade and six patients (60%) improved their KPS score.

### Survival

**Fig. 5:**
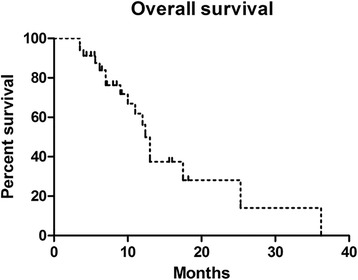
Kaplan-Meier survival curve for all patients with spinal metastases following surgery. Median survival was 12.4 months (95% CI 11.247–13.553)

**Fig. 6:**
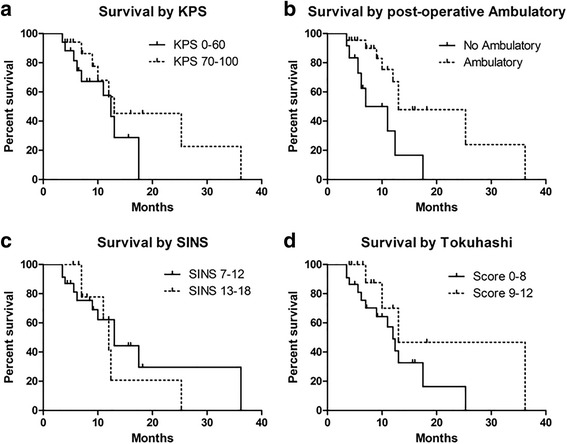
Kaplan-Meier curves for postoperative survival as a function of KPS, ambulatory, SINS, and Modified Tokuhashi score. **a** No difference in survival between patients with preoperative KPS ≥ 70% and KPS < 70% (*P* = 0.631). **b** The survival time was significantly improved for patients with postoperative ambulatory (*P* = 0.009). **c** No difference in survival between patients with preoperative SINS = 7–12 and SINS = 13–18 (*P* = 0.631). **d** No difference in survival between patients with preoperative Modified Tokuhashi score = 0–8 and 9–12 (*P* = 0.122). KPS: Karnofsky Performance Status, SINS: Spine Instability Neoplastic Score

Follow-up ranged from 3.5 to 36.2 months, with an average of 10.2 months, for the whole series. The entire patients’ median survival time after surgery was 12.4 months (range, 3.5–36.2 months, 95% CI 11.247–13.553, Fig. 5). Patients with a preoperative KPS ≥ 70% had a median survival of 13 months (95% CI 0.783–25.217), compared to 12.4 months (95% CI 9.319–15.481) for the KPS < 70% group. However, no significant difference was found on Mantel-Cox testing (HR 0.766, 95% CI 0.259–2.270, *p* = 0.631, Fig. 6a). Notably, patients with nonambulatory that did not improve after surgery had significantly worse median survival (9 months; 95% CI 1.692–12.308) than those who had either no deficit or who regained ambulatory after surgery (13 months; 95% CI 4.197–21.803) (HR 4.888, 95% CI 1.475–16.20, *p* = 0.009, Fig. 6b). Besides, there was no significant difference in median survival when compared to different SINS (SINS 7–12 vs. 13–18, HR 0.766, 95% CI 0.259–2.270, *p* = 0.631, Fig. 6c) or Modified Tokuhashi score (Tokuhashi 0–8 vs. 9–12, HR 2.263, 95% CI 0.803–6.376, *p* = 0.122, Fig. 6d). The median survival of different primary tumors was also analyzed, but no significant difference was detected (*p* = 0.2533, Table 4).

**Table 4:** Primary tumors and median survival time

Primary tumor	*N* (%)	Median survival (months)
Lung	13 (38.2)	13
Breast	7 (21.9)	25.3
Prostate	6 (17.6)	11
Gastrointestinal	2 (5.9)	5.6
Other	6 (17.6)	10

### Correlation between VAS and KPS scores

The Pearson correlation between VAS and KPS scores were further analyzed at different time points. However, the results did not show any significant correlation. The detailed results are shown in Table 5.

**Table 5:** Pearson correlation between VAS and KPS score

Time point	Pre-op	1 month post-op	3 month post-op	6 month post-op	1 year post-op
*r*	− 0.072	− 0.097	0.038	− 0.062	0.069
*P*	0.686	0.585	0.830	0.779	0.840
N	34	34	34	24	11

### Complications

Surgical complications were documented in four patients (11.8%), with two (5.9%) requiring reoperation. One patient (2.9%) experienced deep wound infection after posterior surgery which was successfully treated by surgical debridement. One patient (2.9%) experienced acute epidural hematoma after posterior surgery which was treated by surgical treatment with no neurological deterioration. One patient (2.9%) had cardio-respiratory worsening following anterior or combined posterior-anterior surgery. One patient (2.9%) had postoperative cerebrospinal fluid leakage/effusion confirmed by MRI, whereas 1 year follow-up did not show any special symptom. There were no instrumentation failures in this study.

## Discussion

In recent years, multiple studies have highlighted the effectiveness of surgical treatment for patients with spinal metastases [12–14]. Patchell et al. [13] reported that decompressive surgical resection treatment is superior in regaining ambulatory function to treatment with radiotherapy alone (median 122 vs. 13 days), as well as prolonging survival time (median 126 vs. 100 days) for patients with spinal cord compression caused by metastatic cancer. The better survival time for patients with surgery was probably because a greater proportion of patients were remained or regained ambulatory than those with radiation therapy. Rades et al. [14] reported that motor function and overall survival at the end points after decompressive surgery + radiotherapy displayed better results than those after radiotherapy alone, but there was no significant difference. Surgery allows most patients to remain ambulatory for the remainder of their lives, whereas patients treated with radiation spend a substantial proportion of their remaining time paraplegic. Surgical treatment also reduces the need for corticosteroids and opioid pain relief [13]. However, radiation therapy remains an option for tumor control in patients who choose not to have surgery or are deemed not to be suitable surgical candidate or supplement. There may also be a role for radiation therapy following decompressive surgery [15]. The results in our study shows a median postoperative survival time of 12.4 months and 44% neurological improvement, which highlights the beneficial effects of surgery for patients with spinal metastases.

During the decision-making process of spinal metastases, primary tumor is an important predictive factor. Primary tumor has been included in most of the prognostic scoring systems, e.g., most commonly used Tokuhashi and Tomita scores. These scores have included the effects of primary tumors in predicting survival and making surgical plan. Recently, Bollen et al. [16] has assessed the predictive value of six prognostic scoring systems for spinal metastases based on a retrospective study of 1379 patients. In this study, the percentage and median survival of most prevalent primary tumors were also presented: breast cancer (28%, 18.6 months), lung cancer (23%, 2.0 months), prostate cancer (19%, 7.4 months), kidney cancer (7%, 7.8 months), and colon cancer (5%, 3.1 months). The results of this study indicated that survival time could be varied in different primary tumors. Our results did not show any significant difference in survival time in different primary tumors, which may due to the relative small data size. However, in our clinical experience, patients of spinal metastases with breast cancer usually do better than other primary tumors (including lung or gastrointestinal cancer).

The CTJ site accounts for approximately 10% of all spinal metastases [6]. The most common primary cancers include lung, breast, and prostate cancer [4]. However, spinal metastases involving the cervicothoracic junction and surgical treatment to this region have been sparsely described in the literature [17, 18]. Mazel et al. [18] retrospectively reviewed 32 patients who underwent posterior fixation for cervicothoracic junctional tumors (30 metastasis, 1 chondrosarcoma, and 1 myeloma); the average survive time for patients with vertebrectomy and palliative decompression was 16 (range 3–54) and 11 (range 5–19) months respectively. In the current study, we retrospectively analyzed the surgical outcomes of 34 patients with cervicothoracic junction metastases. Compared with previous studies, the results in this study indicate an acceptable median postoperative survival time (12.4 months) and neurological improvement (44% improved, 56% remained stable). Moreover, the KPS and VAS scores were also significantly improved after surgical treatment. The follow-up data in this report is encouraging and likely reflects the comprehensive effects of prompt and aggressive surgery, radiation, and improved systemic therapies.

In accordance with previous study [13], differences in postoperative ambulatory function showed predictive value for postoperative survival in this study. Neurological impairment in CTJ metastases is favored by narrowing of the spinal canal or vascular damage of the spinal cord, which would deteriorate rapidly in a short period [19]. If the compression is of short duration, the effects are reversible [20]. However, secondary vascular injury occurs with prolonged compression; recovery would be impossible [20]. Thus, prompt surgical involvement is of great importance for CTJ metastases especially with spinal cord compression. The groups were similar in preoperative SINS, Tokuhashi, and KPS scores, and it is possible that the survival difference was not significant due to the small sample size.

The cervicothoracic spine is a junctional area of the vertebral column with its own unique anatomic and biomechanical characteristics [21–23]. Progression from cervical lordosis to thoracic kyphosis at C7-T1 results in transfer of weight from the posterior aspect to the anterior aspect of the spinal column [17, 18]. Furthermore, metastases involving this junctional area are prone to segmental instability, which can lead to excessive kyphosis with subsequent narrowing of the spinal canal and injury to the spinal cord [18, 23]. Therefore, surgical treatment for patients with the CTJ metastases requires proper opportunity and represents unique challenges.

Anterior, posterior, or combined reconstruction can provide stabilization, either as part of a palliative procedure or curative procedure [17]. In the current study, surgical strategy was determined depending on the involved level, tumor location, and the patient’s condition. Anterior approach, specially to the cranial region of the thoracic spine (T1-T4), is technically demanding and not risk-free [24]. Further, a recent study reported that patients with T1 lesions showed biomechanical failure of the anterior construct and subsequently underwent posterior fixation [25]. Thus, anteriorly located cervical lesions were chosen for the anterior approach alone. In this study, 23.52% (8/34) of patients underwent anterior surgery; one patient (12.5%) had cardio-respiratory worsening which was successfully treated.

Posterior fixation techniques have been demonstrated as ideal methods of stabilization for CTJ instability associated with spine tumors [18]. It is especially important to avoid anterior approach in cases in which there is potential posterior ring disruption or pedicle lesion. Therefore, in this series, posterior approach was used for posteriorly located tumors, circumferential lesions, or in regions that were anatomically difficult to access anteriorly. The C7 screw placement is somewhat unique and is considered separately [21–23]. Transpedicular C7 screw insertion is associated with an increased risk of nerve injury due to the unique anatomical morphology and difficulty in achieving clear intraoperative images [21]. In this study, we recommended a pedicle entry point for the C7 pedicle 1 mm inferior to the mid portion of the facet joint with 25° to 30° medial angulation and perpendicular to the posterior arch as previously described [22]. In our series, 64.7% (22/34) of patients underwent posterior surgery. One patient had deep wound infection which was successfully treated by surgical debridement. Another patient had acute epidural hematoma 6 h after surgery, which was manifested as rapid muscle strength worsening confirmed by MRI. Immediate surgical debridement and decompression were performed, and the patient was recovered with no neurological deterioration. This case may indicate that aggressive surgical treatment could save the neurological function for patients with acute epidural hematoma.

Combined posterior-anterior approach was indicated in lesions involving the vertebral body or preexisting spinal deformity [23]. However, surgical risk, patients’ general status, and life expectancy must be evaluated. 11.8% (4/34) of patients underwent posterior-anterior approach surgery. One of them (2.9%) had post-operative cerebrospinal fluid leakage/effusion confirmed by MRI, whereas 1 year follow-up did not show any special symptom. There were no instrumentation failures in all patients.

Indeed, tumor resection and surgical reconstruction in the cervicothoracic junction (CTJ) area pose challenges; the exact surgical indications for different approach are controversial. Kulkarni et al. indicated that posterior fixation is a gold standard for the treatment of cervicothoracic instability in spine tumors, considering anatomic and biomechanical goals [7]. However, other researchers suggested that the surgical approach should be individualized depending on tumor location and involved site [13, 25]. Similarly, in our study, surgical indications are determined for each patient, based on the concept to provide immediate direct decompression and spinal stability reconstruction in the CTJ area. As the follow-up outcomes are acceptable and relatively satisfied, we hope the surgical indications and strategies proposed in this study could provide helpful information.

We have also analyzed the Pearson correlation between VAS and KPS scores. However, no significant correlation was detected, which may partly due to the relatively small data size. The clinical evaluation indexes such as VAS, ODI, and SF-36 are very important and effective to assess the post-operative outcomes for the spinal metastasis patients. They have different features and priorities: VAS is a visual and liner parameter to evaluate neck or back pain, the ODI is a sensitive measure for spinal disorders because it includes domains that are specific to patients with back pain-related disability [26], and SF-36 is a generic multidimensional scale [27], which is reliability and validity for use in the general population and in patients with symptoms of back pain and sciatica with and without surgical intervention. To further clarify the relationship between different indexes, properly designed and larger cohort studies will be needed in the future. Besides, it would also be of great importance to explore a more scientific, effective, and feasible evaluation system for the patients with spinal disorders.

There are some limitations to this study. First, this study is a retrospective research of a selected cohort. Future studies should aim to enroll prospective control clinical trials, in order to determine best practices and guide clinical decision-making. Indexes such as ODI were not included, which should be a limiting factor of this study. Involvement of different companies’ implants and different surgeons in each surgery would have confounding impacts to our study. Further, the overall patient number is small and may be underpowered for statistical significance in some variables due to the small patient cohort. The power of this study is also limited by the use of patients at a single institution and within a restricted time period.

Remarkably, the socio-economic effect is an important dilemma for the surgeon and the patient. The socio-economic condition of each patient must be taken into consideration before surgery. Firstly, strict surgical indications (based on spinal metastasis related evaluating systems, especially significant neurological deficits or intractable pain) must be evaluated for each patient before surgery. Patients with an estimated short survival or poor health situation will be excluded. In this series, most of the patients need long-term use of analgesic drugs or even additional care from their family. It will lead to heavy social and economic burden on the patients, families, and communities. Thus, the aims of the surgery are to improve the function, prevent drug use, maintain the dignity of life, enhance the self-care ability, and provide opportunity for further comprehensive treatment of primary tumor. After surgery, necessary rehabilitation including physiotherapy, electrotherapy, and functional exercise will be given to the patients to promote their recovery and improve their functional status. From our point of view, the decision of a such surgery requires careful consideration based on the strict individualized evaluation and the socio-economic effect for each spinal metastasis patient.

## Conclusions

Based on the results of our study, surgical treatment is effective for patients of CTJ metastases, with a tolerable rate of complications. Furthermore, surgery is associated with improvement in the neurological and overall functional status, as well as alleviation of pain. Remained or regained ambulatory status predicted overall survival. Thus, prompt and aggressive decompressive surgery is recommended for CTJ metastases patients with neurological impairment.

## Abbreviations

CI: Confidence intervals
CT: Computed tomography
CTJ: Cervicothoracic junction
HR: Hazard ratios
IOM: Intra-operative neuromonitoring
KPS: Karnofsky Performance Status
MRI: Magnetic resonance imaging
SINS: Spine Instability Neoplastic Score
SSEPs: Somatosensory-evoked potentials
tcMEPs: Transcranial motor-evoked potentials
VAS: Visual analog scale
